# Tularemia above the Treeline: Climate and Rodent Abundance Influences Exposure of a Sentinel Species, the Arctic Fox (*Vulpes lagopus*), to *Francisella tularensis*

**DOI:** 10.3390/pathogens12010028

**Published:** 2022-12-24

**Authors:** Kayla Buhler, Émilie Bouchard, Stacey Elmore, Gustaf Samelius, Jessica Jackson, Matilde Tomaselli, Heather Fenton, Ray Alisauskas, Emily Jenkins

**Affiliations:** 1 Department of Veterinary Microbiology, Western College of Veterinary Medicine, University of Saskatchewan, 52 Campus Drive, Saskatoon, SK S7N 5B4, Canada; 2 Research Group on Epidemiology of Zoonoses and Public Health (GREZOSP), Faculty of Veterinary Medicine, Université de Montréal, Saint-Hyacinthe, QC J2S 2M2, Canada; 3 Division of Natural Sciences, University of Maine Fort Kent, 23 University Drive, Fort Kent, MA 04743, USA; 4 Snow Leopard Trust, 4649 Sunnyside Ave North, Suite 325, Seattle, WA 98103, USA; 5 Canadian High Arctic Research Station, Polar Knowledge Canada, 1 Uvajuq Road, P.O. Box 2150, Cambridge Bay, NU X0B 0C0, Canada; 6 Government of The Northwest Territories, Department of Environment and Natural Resources, 5th Floor Scotiabank Centre, P.O. Box 1320, Yellowknife, NT X1A 2P9, Canada; 7 Australian Wildlife Health Registry, Taronga Zoo, P.O. Box 20, Mosman, NSW 2088, Australia; 8 Prairie and Northern Wildlife Research Centre, Wildlife Research Division, Environment and Climate Change Canada, 115 Perimeter Road, Saskatoon, SK S7N 0X4, Canada; 9 Department of Biology, University of Saskatchewan, Science Place, Saskatoon, SK S7N 5E2, Canada

**Keywords:** *Francisella tularensis*, tularemia, arctic, rodents, vectors, arctic fox, zoonoses, climate change

## Abstract

Tularemia is a zoonotic disease found throughout most of the northern hemisphere that may experience range expansion with warming temperatures. Rodents and lagomorphs are reservoirs for the disease, and outbreaks of tularemia often follow peaks in their abundance. As small mammals dominate the diet of arctic foxes (*Vulpes lagopus*), we determined whether they may serve as sentinels by identifying antibodies in live-captured and harvested foxes from northern Canada. Overall seroprevalence was 2% (CI95 1–2%) in 176 foxes harvested in 2018–2019 compared to 17% (CI95 12–22%) of 230 foxes captured live in 2011–2021. Prevalence was at an all-time high in 2018, following a peak in vole abundance in 2017. Antibodies were identified in fox pups born in 2018 and 2019, suggesting that *F. tularensis* was actively transmitted during the summers. High precipitation during the summer, increased snow cover and colder temperatures in May, and a higher abundance of voles were all associated with increased seroprevalence in live-captured foxes. Thus, exposure to *F. tularensis* is largely mediated through climate and rodent populations in the Canadian Arctic, and arctic foxes are useful sentinels for *F. tularensis* in northern ecosystems. Further studies should investigate whether infection impacts arctic fox survival and reproductive success in the circumpolar North.

## 1. Introduction

Tularemia is a zoonotic disease of the northern hemisphere caused by the Gram-negative intracellular bacterium, *Francisella tularensis* [1]. Transmission occurs through several routes, including direct contact with infected host fluids, vector bites (mechanical or biological transmission), and through ingestion of contaminated food and water [2]. *Francisella tularensis* is renowned for its high infectivity (as little as 10 colony-forming units) and wide range of hosts and vectors [1]. The source of exposure typically determines clinical symptoms of tularemia that are observed for humans, while the subspecies determines the severity of disease [3]. Ulceroglandular and glandular tularemia, represented by an ulcer and/or swelling of lymph nodes, are the most common forms of disease and occur after bacteria are introduced in the skin via a cut or insect bite. Other forms of the disease include oculoglandular (introduction of bacteria in the eyes), oropharyngeal (ingestion), typhoidal (systemic infection), and pneumonic tularemia (inhalation) [1]. The number of subspecies of *F. tularensis* is debated, but two are known to occur throughout North America. Type A (subsp. *tularensis*) is highly virulent and is associated with terrestrial rodents and lagomorphs, while type B (subsp. *holarctica*) has a circumpolar distribution and is associated with water-dwelling rodents and arthropod transmission (such as mosquitoes, biting flies, and ticks) [1,2,4]. Rodents and lagomorphs (hares and rabbits) are the main reservoirs for aquatic and terrestrial cycles of tularemia transmission, and infection often results in high-mortality events [5,6]. Life history traits, such as population turnover and reproductive output, influence the ability of a reservoir to maintain a pathogen, and those with high population turnover often act as reservoirs for more virulent pathogens [7]. Both rodents and lagomorphs are r-selected species, with high reproduction rates and short lifespans, making them optimal hosts for *F. tularensis* [7].

Across the circumpolar Arctic, rodents and lagomorphs exhibit cyclical population irruptions [8]. These cycles are not fully understood, though mechanisms behind these cycles may include (i) predation (top-down regulation), (ii) social interactions and dispersal (bottom-up regulation), and (iii) effects of climate variability [9]. As the Canadian Arctic is experiencing unprecedented climate change, both increasing temperature and precipitation are likely to create ideal scenarios for tularemia outbreaks in the north [10,11]. Snow depth and the quality of vegetation during the summer and winter months are linked with rodent survival and highlight the influence of weather changes on population irruptions [10,12]. In addition, warming temperatures and increasing precipitation trends provide more opportunities for insect-borne and water-borne transmission by increasing the availability of aquatic habitats and breeding sites for mosquitoes, along with extending the season of mosquito activity [11,13]. *Francisella tularensis* is known to occur in wild animals from most provinces and territories in Canada, yet no studies have documented exposure in wildlife above the treeline [14]. However, one human case (thought to have originated from an insect bite) has been reported in Nunavut, and DNA from *F. tularensis* has been detected in wild-caught mosquitoes from Alaska (Fairbanks), indicating that *F. tularensis* is likely endemic in the north and present in the Canadian Arctic [15,16].

In addition to the potential effects of climate volatility on *F. tularensis* transmission in the North American Arctic, this region also receives millions of migratory birds that may play a role in dissemination of the bacteria when they make their way to breeding colonies from southern overwintering grounds [17,18]. There are at least 26 species of birds known to be susceptible to *F. tularensis* infection, and cases of direct transmission from birds to humans has been documented [19,20]. Transportation of *F. tularensis*-infected arthropods has also been documented on migratory birds [21]. Thus, bird migrations to Arctic breeding grounds may provide opportunities for the dispersal of infected arthropods and the introduction of new subspecies from southern latitudes. 

**Figure 1:**
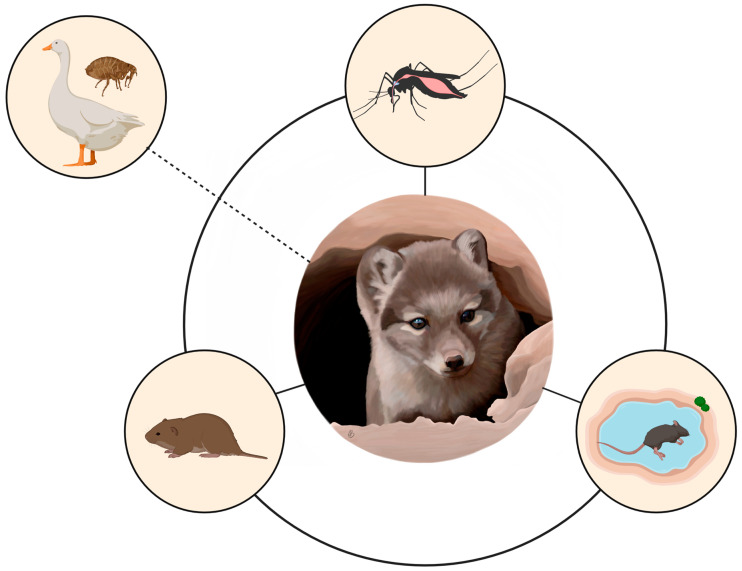
Hypothesized sources of *F. tularensis* transmission for arctic foxes in the circumpolar North. Rodents, insects, and contaminated environments are already associated with transmission in northern ecosystems [16], and as the climate continues to warm, migratory birds and their associated ectoparasites may play a larger role in the dilution or amplification of *F. tularensis* in the Arctic. Created with BioRender.com.

As high mortality is often observed in rodents and lagomorphs during tularemia outbreaks, we hypothesized that scavengers and predators, such as the arctic fox (*Vulpes lagopus*), may serve as important sentinels for *F. tularensis* in tundra ecosystems [22] (Figure 1). Foxes are also important predators of migratory birds and their eggs during summer months [18]. Our study provides baseline information about *F. tularensis* exposure in arctic foxes sampled (lived-trapped or harvested) between 2011 and 2021 within a vast area of the Northwest Territories (NT) and Nunavut (NU) in the Canadian Arctic. Locally intensive vector, rodent, and fox sampling at a long-term field site (Karrak Lake, NU) uniquely allowed us to answer specific questions about *F. tularensis* epidemiology in the Arctic. Our overall project objectives included: (i) determining seroprevalence of *F. tularensis* in arctic foxes from the Canadian Arctic, (ii) determining whether *F. tularensis* DNA was present in insects, migratory geese, and rodents at our long-term study site (Karrak Lake, NU) to identify sources of transmission, (iii) establishing whether climate variables, such as spring snow cover and summer precipitation, and prey variables, such as rodent and goose density, influenced *F. tularensis* exposure of adult and juvenile live-captured foxes at Karrak Lake, and (iv) comparing maternal serostatus with serological results from pups (6–9 weeks of age) to determine if juvenile foxes may serve as seasonal indicators of *F. tularensis* transmission during summer months.

## 2. Materials and Methods

**Figure 2:**
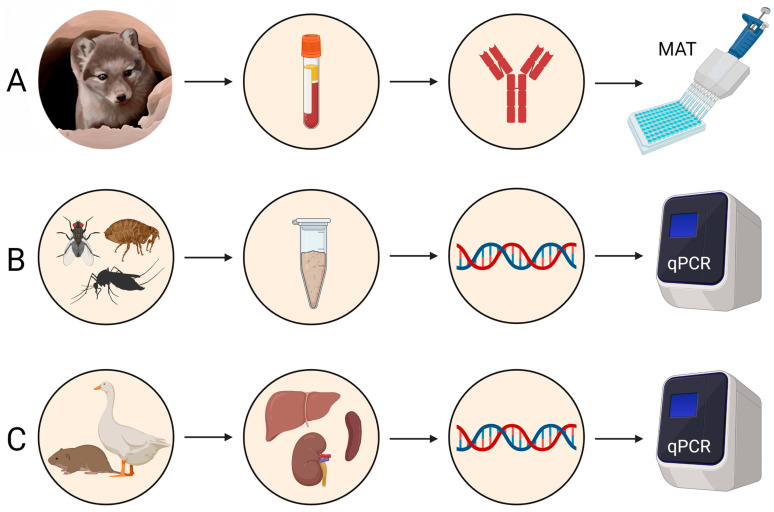
Flowchart of methods. (**A**) Blood was opportunistically collected from both harvested and live-captured foxes and centrifuged to collect serum (live-captured) or supernatant (harvested), and a microagglutination test was used to detect antibodies against *F. tularensis*. (**B**) Insects were collected and pooled per host or per sampling session, and DNA was extracted and subjected to qPCR to detect *F. tularensis* DNA. (**C**) Organs from geese (spleen) and rodents (liver, lung, spleen, and kidney) were collected, DNA was extracted, and samples were subjected to qPCR to detect *F. tularensis* DNA. Created with BioRender.com.

A flowchart of the methods used in this study is provided in Figure 2.

### 2.1. Study Area

**Figure 3:**
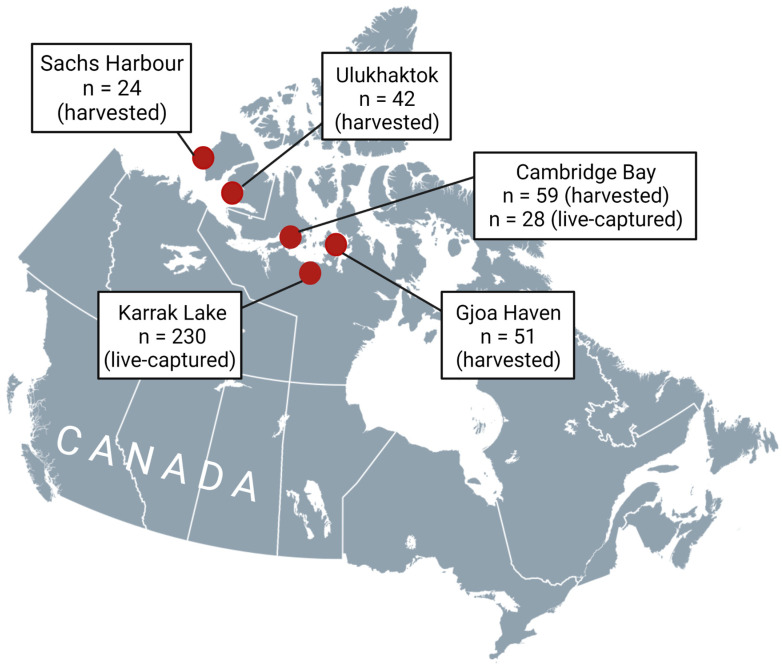
Sampling sites for harvested and live-captured foxes in the Canadian Arctic. Created with BioRender.com.

This study is based on opportunistic sampling of live-captured foxes from Karrak Lake (67°14′ N, 100°15′ W) and Cambridge Bay (69°07′ N, 105°03′ W) in Nunavut, Canada, and harvested foxes from Cambridge Bay, Gjoa Haven (68°38′ N, 95°52′ W), Sachs Harbour (71°59′ N, 125°15′ W), and Ulukhaktok (70°44′ N, 117°46′ W). The work at Karrak Lake, which is in the Queen Maud Gulf (Ahiak) Migratory Bird Sanctuary, was conducted from 2011 to 2019. This sanctuary supports roughly 90% of the world’s population of Ross’s geese (*Anser rossii*) and 15% of the population of lesser snow geese (*Anser caerulescens*) in the summer [23]. The SARS-CoV2 pandemic prevented operations at the Karrak Lake field site during the 2020 and 2021 field seasons. Live capture of adult and juvenile arctic foxes resumed around Cambridge Bay (located on Victoria Island approximately 250 km northwest of Karrak Lake) in 2021. Sampling sites are provided in Figure 3. 

### 2.2. Sample Collection

#### 2.2.1. Arctic Foxes 

Arctic foxes were live-captured during the summers of 2011–2019 and 2021 at Karrak Lake and Cambridge Bay, Nunavut. Briefly, adult foxes (n = 121) were caught in baited box traps and sedated with 0.15–0.20 mL of Telazol^®^ administered intramuscularly [24]. The same method was used to capture pups (n = 109); however, sedation was not necessary. Blood was collected (cephalic or jugular vein) and ear tags were placed in both ears for future identification. Following centrifugation, sera were stored in freezers at −20 °C until tested. In addition, heart blood was collected from arctic foxes that had been harvested for fur by trappers in Cambridge Bay (Iqaluktuuttiaq; n = 59), Gjoa Haven (n = 51), Sachs Harbour (n = 24), and Ulukhaktok (n = 42) during the winters of 2018, 2019, and 2020. When possible, for both live-captured and harvested foxes, weight and sex were determined, a body condition score was assigned (between 1–5 based on fatness), and age was estimated based on tooth eruption and wear [25,26]. During live-capture, we also determined whether foxes were breeding if they were trapped at active den sites with entrances that were kept open and if females were lactating or pregnant. Breeding status was also confirmed later in the summer by the presence of pups at den sites. 

#### 2.2.2. Insects and Geese 

Mosquitoes (multiple species of the Aedes genus) were collected at Karrak Lake during July 2018 using 18 sampling sessions that consisted of 10 rounds of 10 figure eight motions with an 18-inch sweep net. In between rounds, insects were collected with an aspirator and, following each sampling session, mosquitoes were frozen in plastic containers at −20 °C for 24 h. Mosquitoes and other insect bycatch were then placed in petri dishes with a single tissue to ensure safe transportation and were kept at −20 °C until they were identified. 

Avian fleas (*Ceratophyllus vagabundus vagabundus*) were collected from incubating nests (n = 24) at Karrak Lake during the summer of 2019 with a 25×25 cm square of white flannel as per Harriman et al. [27], placed into individual Ziploc bags, and frozen overnight. Once the fleas were dead, they were collected from the flannel, pooled per nest, and placed in microcentrifuge tubes with 70% ethanol (up to 5 fleas per tube). Similarly, avian fleas were collected from the carcasses of Ross’s (n = 42) and lesser snow geese (n = 40) that were collected upon arrival at the colony in late May 2019. Immediately following collection, goose carcasses were placed in clear plastic bags and held at ambient temperature for 24 h. Fleas were then collected from the bags and carcasses, pooled per host, and placed in microcentrifuge tubes containing 70% ethanol (maximum of 5 fleas per tube). Spleens were also collected from these geese and frozen until tested. 

#### 2.2.3. Rodents

Rodents have been trapped at Karrak Lake via line transects (3 transects placed in the same locations each year containing 25 snap traps checked for 10 days) with museum snap traps for more than 20 years to estimate abundance [28]; however, rodent carcasses were only collected for testing during the summer of 2019. Most Arctic rodents spend their time under the snow during winter; thus, it is not possible to snap-trap outside of the summer season. The line transects were checked daily and rodent carcasses were placed individually into Ziploc bags and frozen at −20°C. Rodents were identified as northern collared lemmings (*Dicrostonyx groenlandicus*) and northern red-backed voles (*Myodes rutilus*). A sample of liver, lung, spleen, and kidney was collected from each individual and pooled in 1.5 mL Eppendorf tubes (Thermo Fisher Scientific, Waltham, MA, USA). 

All wildlife and insect samples were sent to the Zoonotic Parasite Research Unit (Western College of Veterinary Medicine, Saskatoon, Canada) and stored at −20 °C until tested. 

### 2.3. Microagglutination Assay to Detect Antibodies for F. tularensis 

A microagglutination assay (MAT) was used to detect IgM and IgG antibodies for *F. tularensis* in serum samples collected from live-captured and harvested foxes [29]. Human and arctic fox sera that were previously tested with a MAT were used as positive and negative controls (human sera provided by National Microbiology Laboratory, Winnipeg, Canada). A high positive control (1:1024), low positive control (1:128), and negative control were used during each run. Briefly, 25 μL of microagglutination buffer (phosphate buffered saline with 1% normal rabbit serum and 0.4% formalin) was added to the wells of round-bottom 96-well plates. Serum samples were serially diluted across each row by mixing 10 times and transferring 25 μL to the following well with a multichannel pipettor. The remaining 25 μL from the final row was discarded. Next, 25 μL of antigen (formalin-killed *F. tularensis* cells) was added to the wells. Each plate was then covered with plastic wrap and incubated for 24 h at room temperature. Wells were visually inspected for agglutination, and titers ≥ 1:128 were considered positive.

### 2.4. DNA Extraction and Quantitative PCR for F. tularensis

The RNeasy Mini Kit (as per manufacturer specifications; Qiagen; Hilden, Germany) was used for mosquito pools, as RNA was also required for further disease testing. DNA was extracted from pooled rodent samples (liver, lung, spleen, and kidney), avian fleas, and black flies using the DNeasy Blood & Tissue Kit (Qiagen) as per manufacturer specifications. All samples were checked with NanoDrop™ (Thermo Scientific; Waltham, MA, United States) to ensure sample quality and presence of DNA prior to proceeding with real-time reverse transcriptase-polymerase chain reaction (RT-PCR). RT-PCR was performed on samples using primers TUL936 (5′-CCG CTA CAG AAG TTA TTA CCT TGC T-3′) and TUL841 (5′-CCA TGA TAC AAG CTT CCC AAT TAC T-3′). The probe was TUL4 871 (6FAM- TGC TGA GAA GAA CGA TAA AAC TTG GGC AAC -TMR). RT-PCR was conducted using the following conditions: a hold stage of 50 °C for 2 min and 95 °C for 10 min followed by 40 cycles of 95 °C for 15 s and 60 °C for 1 min. A 30 µL reaction mixture was used containing 5 µL SsoAdvanced Universal Probes Supermix (Bio-Rad Laboratories; Hercules, United States), 11.4 µL H2O, 0.5 µL of each primer (20 µM), 0.15 µL of probe (25 µM), and 5 µL of template. Positive controls were gBlocks™ gene fragments (Integrated DNA Technologies, Coralville, United States) created from the complete genome of *F. tularensis* subsp. *holarctica* reported in GenBank (Accession number CP044005.1).

### 2.5. Insect Identification

Flea species were morphologically identified as described by Holland [30]. Mosquitoes were morphologically identified to the genus level as described by Thielman and Hunter [31]. Black flies were collected as bycatch from mosquito sampling efforts but were not identified. DNA was extracted from flea pools using the DNeasy Blood & Tissue Kit (Qiagen Inc; Hilden, Germany). Conventional PCR targeting ~615 bp of the mitochondrial cytochrome c oxidase II gene (COII) was conducted using the primers COII-2a (5′-ATA GAK CWT CYC CHT TAA TAG AAC A-3′) and COII-9b (5′-GTA CTT GCT TTC AGT CAT CTW ATG-3′) [32]. PCRs were conducted as per Buhler et al. [33]. Samples that successfully amplified were then purified using the QIAquick PCR Purification Kit (Qiagen Inc. Hilden, Germany) and sequenced (Macrogen; Seoul, South Korea).

### 2.6. Statistical Analysis 

*Francisella tularensis* seroprevalence of live-captured and harvested foxes along with 95% confidence intervals (CI95) were calculated using EpiTools epidemiological calculators [34]. Associations between biological variables (age, sex, weight, body condition score, and sample type (whole frozen heart blood or serum)) and exposure to *F. tularensis* (positive MAT result) were evaluated for live-captured and harvested foxes by using linear regression. In addition, associations between climate variables and exposure to *F. tularensis* were evaluated via stepwise linear regression for both the current year and the year prior to capture for adult and juvenile foxes from Karrak Lake (2011–2019). Climate variables included average temperature for May, June, July, and August (°C), average snow cover in May (cm), and total precipitation from May to August (mm). Climate data were extracted from the North American Regional Reanalysis (NARR) using Env-Data (hosted by Movebank) for the Karrak Lake coordinates (67.233, −100.25). Finally, associations between prey variables, including rodent abundance (number of lemming captures per 100 trap nights and number of northern red-backed vole captures per 100 trap nights), goose density in the colony (total abundance of white geese divided by the square kilometers occupied by the colony), and fox exposure to *F. tularensis* in the current year and year prior to trapping, were evaluated using stepwise linear regression. Goose density was calculated from an aerial survey of the Karrak Lake colony during June of each year, while rodent abundance was estimated from captures on three traplines placed near Karrak Lake in the same locations each year. As the same adult foxes were often sampled over multiple years at Karrak Lake, and each year’s titer was considered independently in the above models (there is no knowledge of the duration of antibody production against *F. tularensis* in arctic foxes); the same analyses identifying associations between (1) climate variables or (2) prey variables and *F. tularensis* exposure in fox pups (2014–2016 and 2018–2019) were completed to eliminate any effects that may have been observed due to repeat sampling of adults. Statistical analyses were conducted in SPSS (Version 28; IMB Corporation 2021).

## 3. Results

All avian fleas were identified morphologically and molecularly as *Ceratophyllus vagabundus vagabundus* (100% identity: QNU09891). Mosquitoes were identified morphologically as black-legged species of the Aedes genus, including *Aedes nigripes*, *Aedes hexodontus*, and *Aedes impiger*. Pooled flea samples (*C. v. vagabundus*) from nests (n = 59, up to five fleas per pool), and geese (n=35, up to five fleas per pool), mosquitoes (n = 1163; 16 pools of up to 73 mosquitoes), black flies (n = 66; 14 pools of five flies), geese (n = 82 snow and Ross’s geese), and rodents (13 northern red-backed voles and 8 northern collared lemmings) were all negative for *F. tularensis* DNA. 

### 3.1. Estimated Prevalence of F. tularensis in Arctic Foxes

Overall seroprevalence in foxes was 10% (n = 42/406; CI95 8–14%). Total seroprevalence in harvested foxes was 2% (n = 4/176; CI95 1–2%). Seroprevalence in carcasses was 0% in Ulukhaktok (n = 42) and Sachs Harbour (n = 24) in the Inuvialuit Settlement Region, 5% in Cambridge Bay (n = 3/59; CI95 2–14%), and 2% in Gjoa Haven (n = 1/51; CI95 0–10%). Arctic foxes live-captured at Karrak Lake and Cambridge Bay, Nunavut, had higher prevalence of *F. tularensis* antibodies than harvested foxes. A detailed account of exposure in adults and pups live-captured from 2011 to 2021 is provided in Table 1. 

### 3.2. Factors Influencing Exposure 

Sample type (β = −0.5, CI95 −0.45 to −0.25) was the only statistically significant variable when identifying associations between antibody presence in harvested and live-captured foxes and biological variables (R^2^ = 0.25, df = 5, *p* = < 0.001), with a higher number of positives documented using sera. When investigating associations between prey variables and antibody presence in live-captured foxes at Karrak Lake, only variables for the year prior to sampling were associated with increased exposure, including a higher abundance of voles along with a lower abundance of lemmings and lower goose density (Model 1; Table 2). When identifying associations between climate variables and antibody presence in live-captured foxes at Karrak Lake, more snow cover in May and higher precipitation accumulated from May to August were associated with increased exposure (Model 2; Table 2). In addition, warmer temperatures in May during the year prior to fox sampling were associated with more *F. tularensis* exposure. When data from live captured adults at Karrak Lake were excluded from the analysis (dependent variable was only considered to be antibody presence in live-captured juveniles), colder temperatures in May during the concurrent year were associated with *F. tularensis* exposure (Model 4; Table 2). Finally, similar results were obtained in the model that investigated associations between *F. tularensis* exposure in pups and prey variables, as a higher abundance of voles and a lower abundance of lemmings during the year prior to sampling was associated with *F. tularensis* exposure (Model 3; Table 2). 

## 4. Discussion

We provide the first description of *F. tularensis* in wildlife above the treeline in northern Canada and the factors that impact transmission in tundra ecosystems, which are primarily climate and rodent abundance. Our long-term study of arctic foxes at Karrak Lake presented a unique opportunity to monitor this population over a nine-year period. During 2011–2013, no antibodies for *F. tularensis* were detected in live-captured foxes. However, from 2014–2019, both adult and juvenile foxes were identified with positive titers, ranging from 1:128 to >1:2048. The year with the highest estimated seroprevalence was 2018, following a peak in vole abundance in 2017. This year appeared to be an outbreak year, with high prevalence and antibody titers in both adult foxes and pups (Table 1). In 2019, blood was successfully collected twice from pups at two den sites (n = 11; once at six weeks of age and once at eight weeks of age). During the time between first and second blood collection, three pups developed antibodies for *F. tularensis*, which again supports that the bacteria were actively circulating within the environment during the summer months. The observation of seronegative pups in litters from seropositive breeding females suggests that maternal antibodies did not interfere with test results (Figure S1). In addition, litters often had both negative and positive pups, which indicates that transmission via insects or rodents may be more likely than a common contaminated water source (Figure 1). No visibly ill or dead foxes were observed, even in 2018 and 2019, suggesting that there may have been nonlethal exposure of foxes through rapid consumption of rodents that may have died acutely due to tularemia. Indeed, fox pups might preferentially feed on easy prey such as septicaemic rodents and scavenge their carcasses.

More snow cover in May during the year of live capture of foxes at Karrak Lake was associated with increased *F. tularensis* exposure (Model 2; Table 2). This makes sense, as rodent survival is influenced by spring temperatures, and longer periods of snow cover can reduce predation, leading to higher population density of rodents during the summer [12]. Ironically, warmer temperatures in May during the year prior to sampling was also associated with increased exposure for foxes, potentially due to reduced rodent survival during the spring, which may have created higher resource availability and provided the opportunity for more reproductive success the following winter. Finally, prevalence of *F. tularensis* increased with increasing precipitation in the same year that foxes were captured. Both temperature and precipitation impact vegetation growth, which in turn influences rodent population cycles [9]. For example, higher summer precipitation may lead to higher abundance of berry crops, which is an important food source for northern red-backed voles [35,36]. The availability of food plants with high nutritive value perhaps contributed to high rodent population density over the summer months. 

When adult foxes were excluded from the regression analyses, only colder temperatures in May during the year of sampling remained as an important climate correlate of pup exposure (Model 4; Table 2). This is logical, as colder temperatures should lead to longer snow cover in spring, limiting stress and predation, which should favor reproduction and contribute to higher rodent population density during the summer [37,38,39]. Furthermore, colder springs can induce earlier onset of reproduction for voles and may contribute to a higher population density during the summer months [40].

When examining associations between prey variables (abundance measures collected in June and July) and the presence of *F. tularensis* antibodies, lower abundance of lemmings and higher abundance of voles during the year prior to sampling was associated with higher seroprevalence for both the model that included adult and pup serology results (Model 1; Table 2) and the model that only included pup results (Model 3; Table 2). This corresponds with previous reports of density-dependent effects for *F. tularensis* and rodent populations [41] and highlights that vole abundance may play an important role in the dissemination of *F. tularensis* in this tundra ecosystem. It also supports our hypothesis that higher temperatures in May and potentially lower rodent survival during spring in the year prior to fox exposure (Model 2; Table 2) may have provided an opportunity for increased reproduction during the following summer for northern red-backed voles. 

Both lemmings and voles often coexist by using different microhabitats in tundra environments, with lemmings inhabiting drier habitats and voles inhabiting wetter habitats [42]. The use of different microhabitats limits competition between these rodents, which could minimize transmission risk from one species to another. Given that water-borne transmission has been well-documented for *F. tularensis* and that it can remain viable in cold water (8°C) for at least 70 days, it is therefore not surprising that rodents that occupy wetter environments may play a larger role in transmission of the bacteria [43]. None of the lemmings and voles sampled during this study were actively infected with *F. tularensis*. High mortality in rodents following infection suggests that it is unlikely that infected animals would be collected during snap trapping, as foraging rodents should generally be healthy animals [5]. Our sample size was also quite small (n=21), probably sampling an insignificant proportion of the population. Rodent mortality events may go undetected in tundra ecosystems (large geographic area with little human activity), highlighting the importance of identifying sentinel species for *F. tularensis* that will scavenge carcasses of these small mammals. It is important to note that there are no studies that have investigated lethality following *F. tularensis* infection in Arctic rodents. One study found that rats infected with subsp. *tularensis* (type A) did not survive past 72 h [44]. Documenting mortality for small mammals in the wild is complicated by the fact that scavengers are likely to consume carcasses before humans notice these events, especially in remote locations. However, during population peaks, mortality may be so high that scavengers cannot keep up with the number of dead animals. For example, this was seen during an outbreak of subsp. *holarctica* (type B) in deer mice from Canada [45]. Thus, it is not possible to estimate lemming or vole mortality due to tularemia without studies in controlled environments, and outbreaks may go unnoticed (depending on population density).

Weaned juvenile foxes appear to be ideal sentinels for summer prevalence, and surveillance at den sites around communities may be a useful indicator of seasonal risk for rodent-borne diseases (Table 1). Foxes rely heavily on voles and lemmings as a food source, and they are important predictors for litter size, breeding density, and annual variation in fox abundance [46,47]. Thus, monitoring reproductive success of arctic foxes and identifying years with a higher density of young foxes may provide important information with regards to *F. tularensis* risk in the environment. 

Our study site at Karrak Lake is home to one of the largest white goose colonies in the Canadian Arctic, and foxes in this region rely heavily on the goose population as a dietary source during the summer months [47]. Despite this seasonal superabundance of migratory prey, we identified associations between *F. tularensis* exposure and rodent abundance, suggesting that foxes can still be useful sentinels in regions where they have a diversified diet. Lower goose density during the year prior to fox sampling was associated with *F. tularensis* exposure in the first model that included serology results for all foxes from Karrak Lake (Model 1; Table 2), which is logical, as lower numbers of lemmings and geese during the summer prior to the outbreak year in 2018 would have led to higher consumption of voles and potentially more exposure to the bacteria. Birds can be infected with *F. tularensis*, migratory birds have been implicated in the spread of the bacteria via the transportation of ectoparasites, and direct bird-to-human transmission has been documented [20]. Although birds may play a role in the transmission of *F. tularensis* at Karrak Lake (Figure 1), none of the nest fleas or goose spleens contained *F. tularensis* DNA. In fact, an interesting research question for the future would be if years with higher goose density in the colony might dilute transmission of *F. tularensis* by providing a significant food source for foxes, thus reducing consumption of rodents.

A variety of insects were collected and tested for *F. tularensis* DNA during this study, and none were positive. While sample size was relatively small for fleas and black flies, the sample size was large for mosquitoes (n = 1163). Mosquitoes were collected during the summer of 2018, when we observed the highest seroprevalence of *F. tularensis* in both adult and juvenile foxes. DNA of *F. tularensis* has been identified in mosquitoes from North America (Fairbanks, Alaska); however, these were all collected below the treeline [16]. Our study suggests that vectors may not be significantly involved in the transmission of *F. tularensis* in the Canadian Arctic, though further studies are needed to identify whether insects play a role in transmission of the bacteria above the treeline. 

Serum samples collected from live-captured foxes were significantly more likely to be positive than whole blood collected from carcasses, which may be attributed to three potential reasons. First, this may be due to sample quality, as carcasses are often frozen and thawed several times during skinning and necropsy. Second, this may reflect spatial variation between sample locations, as live trapping at Karrak Lake occurred on the mainland of Nunavut and fox carcasses were collected from trappers in communities around the Arctic Archipelago. Though foxes can disperse across long distances and over the Arctic Sea ice [48], the absence of voles on the islands may have contributed to the low seroprevalence observed in fox carcasses. Lemmings are generally the only rodents collected during snap-trapping efforts on these islands (*Dicrostonyx* and *Lemmus*), while both lemmings and voles are present in ecosystems further south, such as Karrak Lake [18,49]. There are differences between these two groups of rodents in their habitat use (voles are often associated with aquatic habitats) and other life history traits that influence their reservoir potential for *F. tularensis* [7]. Third, the timing of the trapping season may also impact the number of positives observed, as trappers typically wait until foxes have a full winter coat before setting out traplines (from November to March). As *F. tularensis* can be transmitted via arthropods or ingestion of contaminated water sources, freezing temperatures during the trapping season in the Arctic minimizes these sources of transmission (Figure 1) [2]. The potential for transmission via contact with rodents continues in the winter, though dense snow cover and freezing conditions may reduce the ability of foxes to scavenge rodent carcasses during the high-mortality events that are associated with tularemia epizootics [5]. Serum samples from live-captured foxes at Karrak Lake were collected during late spring and summer (May–July) of each year, which would provide a more favorable environment for transmission. Thus, foxes may be more effective sentinels if samples are collected in the summer vs. winter, especially if antibody production is short-lived. 

## 5. Conclusions

In conclusion, we described the prevalence of *F. tularensis* in arctic foxes from Arctic Canada and identified important associations between climate, rodent abundance (especially voles), and seroprevalence. The Arctic is experiencing accelerated climate change, and both temperature and precipitation have increased over the past 20 years [11]. These trends are predicted to continue across the circumpolar North and will likely impact both rodent communities and transmission of *F. tularensis* as the climate becomes more favorable [11,50]. We were unable to determine if *F. tularensis* type A or B was present in the Karrak Lake ecosystem, as all samples tested with RT-PCR were negative and the MAT did not differentiate between subspecies. Thus, further studies are needed to determine whether type A or B circulate in this ecosystem. Arctic foxes and rodents are closely linked via a strong predator–prey relationship [47,51]. During this close relationship, it is likely that foxes evolved alongside common rodent-borne pathogens, such as *F. tularensis*. During the summers of 2018 and 2019, many pups were identified with titers greater than 1:512, indicating recent exposure. The absence of any observable symptoms or mortalities in litters during these years suggest that foxes may have adapted to tolerate infection with the bacteria, which presents another interesting research question for the future. 

## Figures and Tables

**Table 1:** Prevalence of *F. tularensis* antibodies in live-captured Arctic foxes from central Nunavut. Years when fox pups were not captured are blacked out (either due to resource limitations (2011–2013) or a low number of breeding foxes at Karrak Lake (2017)).

Year	Location	Total Number of Foxes Tested	Total Seroprevalence (%)	CI95 (%)	Total Number of Adults	Adult Seroprevalence (%)	CI95 (%)	Total Number of Pups	Pup Seroprevalence (%)	CI95 (%)
2011	Karrak Lake	10	0	0–28	10	0	0–28			
2012	Karrak Lake	18	0	0–18	18	0	0–18			
2013	Karrak Lake	16	0	0–19	16	0	0–19			
2014	Karrak Lake	32	3 (n = 1)	1–16	14	7 (n = 1)	1–32	18	0	0–18
2015	Karrak Lake	24	4 (n = 1)	1–20	11	9 (n = 1)	2–38	13	0	0–23
2016	Karrak Lake	15	27 (n = 4)	11–52	5	80 (n = 4)	38–96	10	0	0–28
2017	Karrak Lake	4	0	0	4	0	0–49			
2018	Karrak Lake	44	45 (n = 20)	32–60	21	48 (n = 10)	28–68	23	44 (n = 10)	26–63
2019	Karrak Lake	39	21 (n = 8)	11–36	14	29 (n = 4)	12–55	25	16 (n = 4)	6–35
2021	Cambridge Bay	28	14 (n = 4)	6–32	8	50 (n = 4)	22–79	20	0	0–16

**Table 2:** Stepwise linear regression analyses for the assessment of prey variables and climate variables associated with *F. tularensis* antibody presence in arctic foxes at Karrak Lake, Nunavut.

**Model**	Variables ^*^	**B**	**SE**	**β**	**t**	***p***	**95.0% Confidence Interval for B**
							**Lower Upper**
1 ^a^	Constant YP RBV YP Goose YP Lemming	0.377 0.209 −7.43 × 10^−5^ −0.037	0.133 0.053 0 0.013	0.265 −0.199 −0.184	2.839 3.916 −2.932 −2.832	0.005 <0.001 0.004 0.005	0.115 0.639 0.104 0.315 0 0 −0.063 −0.011
2 ^a^	Constant Snow Cover May Total Precip Ac YP Temp May	−0.162 0.004 0.007 0.111	0.237 0.002 0.001 0.028	0.17 0.409 0.372	−0.687 2.16 5.057 3.934	0.493 0.032 0 <0.001	−0.629 0.304 0 0.008 0.004 0.01 0.055 0.167
3 ^b^	Constant YP RBV YP Lemming	0.021 0.292 −0.289	0.068 0.068 0.1	0.408 −0.272	0.31 4.312 −2.878	0.757 <0.001 0.005	−0.113 0.155 0.158 0.427 −0.488 −0.089
4 ^b^	Constant Temp May	−1.046 −0.187	0.307 0.047	−0.39	−3.409 −3.949	<0.001 <0.001	−1.655 −0.436 −0.282 −0.093
**Model**	**R**	**R^2^**	**Adj R^2^**	**SEE**	**R^2^ Change**	**F Change**	**Sig. F** **Change**
1 ^a^	0.408	0.166	0.154	0.345	0.034	8.02	0.005
2 ^a^	0.428	0.184	0.171	0.341	0.064	15.48	<0.001
3 ^b^	0.482	0.232	0.214	0.325	0.074	8.29	0.005
4 ^b^	0.39	0.152	0.142	0.339	0.152	15.59	<0.001

^a^ Model 1 and 2 = dependent variable is the presence or absence of *F. tularensis* antibodies in live-captured adult (>1 year) and juvenile (6–9 weeks) arctic foxes. ^b^ Model 3 and 4 = dependent variable is the presence or absence of *F. tularensis* antibodies in live-captured juvenile (6–9 weeks) arctic foxes. * YP RBV = year-prior northern red-backed vole abundance (vole captures per 100 trap nights); YP Goose = year-prior goose abundance per square kilometer; YP Lemming = year-prior lemming abundance (collared and brown lemming captures per 100 trap nights); Snow Cover May = average snow cover in May (cm) during the year of sampling; Total Precip Ac = total precipitation from May to August (mm) during the year of sampling; YP Temp May = year-prior average temperature for the month of May (°C); Temp May = average temperature for the month of May (°C) during the year of sampling.

## Data Availability

Not applicable.

## Supplementary Materials

The following supporting information can be downloaded at: https://www.mdpi.com/article/10.3390/pathogens12010028/s1, Figure S1: Exposure to *Francisella tularensis* in breeding pairs of arctic foxes and their pups in 2018 and 2019.
