# Supplementary material for: Tularemia above the Treeline: Climate and Rodent Abundance Influences Exposure of a Sentinel Species, the Arctic Fox (Vulpes lagopus), to Francisella tularensis
Source: Pathogens. 2022 Dec 24;12(1):28. doi: 10.3390/pathogens12010028 (PMC9861794; doi:10.3390/pathogens12010028)
Supplement: Supplementary file 1 [file pathogens-12-00028-s001.zip › pathogens-2083483-supplementary.pdf]

Supplemental Figure S1. Exposure to *Francisella tularensis* in breeding pairs of arctic foxes and their pups in 2018 and

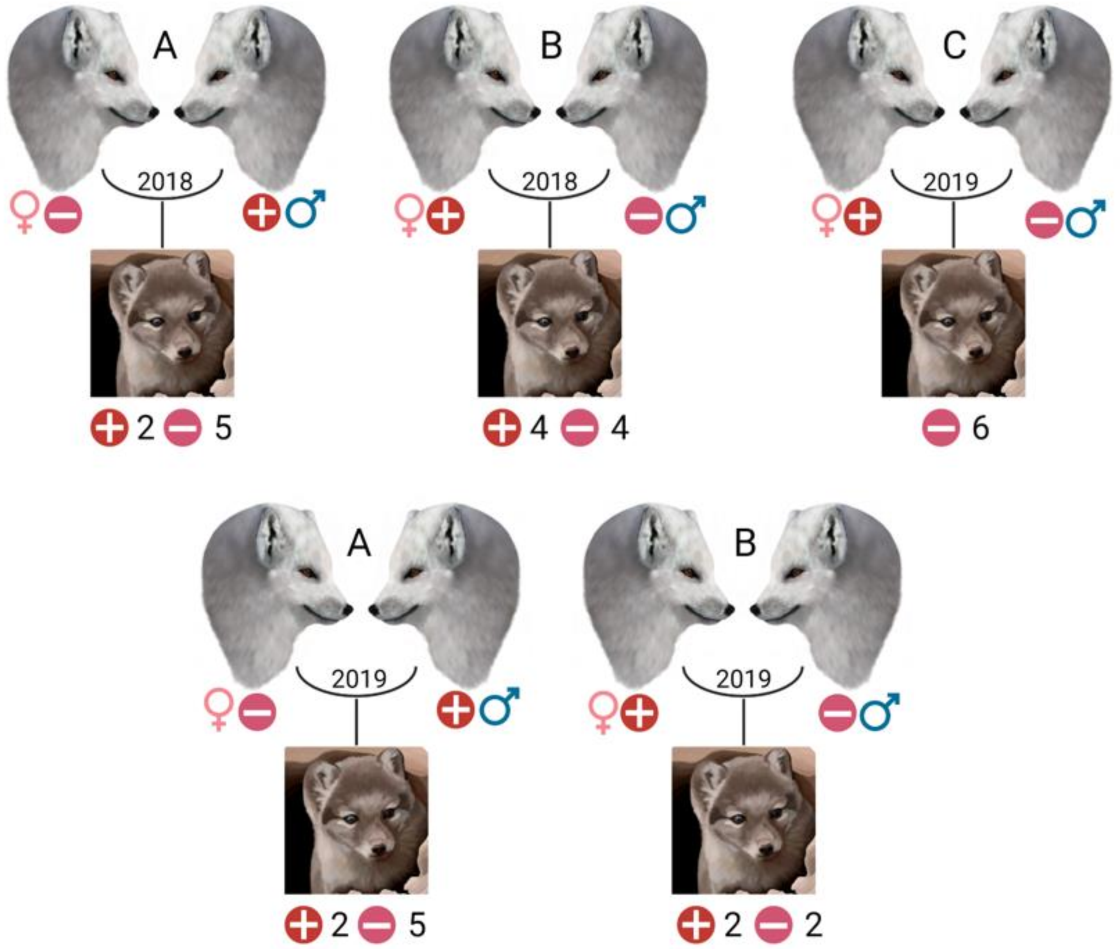

Figure S1. Dens that were tested with unknown status of adults were not included. Both pair A and B were sampled in both 2018 and 2019. Created with BioRender.com
